# Electro-acupuncture on functional peripheral nerve regeneration in mice: a behavioural study

**DOI:** 10.1186/1472-6882-12-141

**Published:** 2012-08-31

**Authors:** Ngoc Son Hoang, Chamroeun Sar, Jean Valmier, Victor Sieso, Frédérique Scamps

**Affiliations:** 1 Inserm, U 1051, Montpellier, F-34000, France; 2 University Montpellier I, Montpellier, F-34000, France; 3 University Montpellier II, Montpellier, F-34000, France; 4 Hanoi Medical University, Hanoi, Vietnam

**Keywords:** Sciatic nerve, Crush, Nerve injury, Pain, Sensorimotor

## Abstract

**Background:**

The improvement of axonal regeneration is a major objective in the treatment of peripheral nerve injuries. The aim of this study was to evaluate the effects of electro-acupuncture on the functional recovery of sensorimotor responses following left sciatic nerve crush in mice.

**Methods:**

Sciatic nerve crush was performed on seven week old female mice. Following the injury, the control group was untreated while the experimental group received an electro-acupuncture application to the injured limb under isoflurane anesthesia at acupoints GB 30 and GB 34. Mechanical and heat sensitivity tests were performed to evaluate sensory recovery. Gait analysis was performed to assess sensorimotor recovery.

**Results:**

Our results show that normal sensory recovery is achieved within five to six weeks with a two-week period of pain preceding the recovery to normal sensitivity levels. While electro-acupuncture did not accelerate sensory recovery, it did alleviate pain-related behaviour but only when applied during this period. Application before the development of painful symptoms did not prevent their occurrence. The analysis of gait in relation to the sensory tests suggests that the electro-acupuncture specifically improved motor recovery.

**Conclusions:**

This study demonstrates that electro-acupuncture exerts a positive influence on motor recovery and is efficient in the treatment of pain symptoms that develop during target re-innervation.

## Background

Traumatism of peripheral nerve and unsuccessful regeneration lead to ataxia as well as the occurrence and maintenance of pain-related behaviour. Therefore, the improvement of axonal regeneration is a major objective in the treatment of peripheral nerve injuries and associated neuropathies. The sciatic nerve crush model is a well-characterized model of peripheral nerve regeneration. After a crush lesion, nerve fibres in the distal stump degenerate and the expression of regeneration-associated genes takes place [1-3]. Presently, very few drugs are available that reliably enhance the rate and completeness of nerve regeneration [4]. Other strategies, such as the utilization of electrical fields, have been explored. Electrical stimulation of the injured nerve with biphasic currents was shown to promote motoneuron regeneration [5-7], but also to increase the intrinsic regenerative capacity of sensory neurons [8]. However, the invasiveness of these techniques has led to the assessment of other methods such as nerve stimulation with a transcutaneous electrical field, which was instead shown to delay nerve regeneration [9]. Electro-acupuncture represents another method with minimal tissue damage and known to produce anti-hyperalgesia in animal models of inflammatory pain. Electro-acupuncture was also shown to improve motor recovery by assessing muscle electrical activity elicited under peripheral nerve stimulation [10]. However, no studies have explored its effects on peripheral nerve regeneration by measuring both motor and sensory recovery.

Although morphological and functional techniques are available to assess nerve fibre regeneration, they all have some limitations. Indeed, there is a lack of a direct relationship between morphological data and functional studies [9,11]. Furthermore, while electrophysiological tests can give important information on the functionality of regenerated axons, they also have methodological limitations [12]. Thus, demonstrating any benefits to the functioning of the axon’s target organ would ensure clinical interest. This can be achieved by using behavioural tests, which provide a valuable functionality index. Automated gait analysis is a non-invasive method allowing analysis of motor and sensory recovery in freely moving, non-restrained animals. This method can simultaneously measure dynamic as well static gait parameters and was also effective in assessing mechanical allodynia [13,14].

In the present study, we used gait analysis and sensory testing to investigate the effects of electro-acupuncture on the functional regeneration of the sciatic nerve after crush injury.

## Methods

### Surgical procedure

This study was performed on 46 adult C57BL/6 virgin female mice (6–8 weeks old, CERJ, Le Genest St. Isle, France). Their oestrous phase is about 12 h every 3–9 days, and the majority of mice are synchronized in our breeding house, so that the observed differences in this study are probably not due to hormonal differences in the two groups. The care and use of mice conformed to institutional policies and guidelines and was approved by the Animal Experimentation Ethics Committee of the University of Montpellier. Mice were housed in cages with a 12 h light/dark cycle and fed food and water ad libitum.

All animals were deeply anesthetized by isoflurane inhalation. Their left gluteal regions were shaved and cleaned with betadine. The left sciatic nerve was exposed at the mid-thigh without any damage to the muscle tissue and a 1 mm length of the sciatic nerve was crushed with Dumont #5 forceps for 15 sec. The animals were randomly divided into two groups. In group 1 there were 16 controls and 10 mice that were electro-acupuncture-treated 3 days after the nerve crush. To investigate the effects of electro-acupuncture during the period of pain-related behaviour (i.e. 15–28 days post-operation), we used a second group of mice, termed group 2, with 10 controls and 10 mice that were electro-acupuncture-treated two weeks after nerve crush. Group 1 was included for sensory tests and CatWalk analysis. Group 2 was assessed only for sensory testing.

### Electro-acupuncture stimulation

The acupoints used were Hoantiao GB30 and Yanglinquan GB34. In humans, GB30 and GB34 are used to treat sciatic nerve pain and paralysis. They have been demonstrated to be analgesic acupoints in animal models of peripheral inflammatory pain [15] and molecular mechanisms involved in the analgesic effects of acupuncture have been postulated to contribute to nerve regeneration [16]. According to World Health Organization standards, GB30 is located in the buttocks region, at the junction of the lateral one third and medial two thirds distance between the prominence of the greater trochanter and the hiatus of the sacrum [17]. In the transpositional animal acupoint system for mouse and rat models, it is located at the depression superior to the greater trochanter of the femur [18]. GB34 is located at the depression anterior and inferior to the fibular head.

For the group receiving electro-acupuncture, the following steps were performed: under isoflurane anaesthesia, electrical stimulation (2 Hz, rectangular pulse, 0.5 ms duration, intensity 0.8- 1 mA for 15 minutes) was applied via two acupuncture needles (diameter 0.2 mm, 2 cm length) inserted to a depth of 3 mm into the acupoints using an electrical stimulator (Improved KWD-808-II apparatus). The intensity of the stimulation was enough to produce a twitching of the hind leg. The low frequency stimulation used in the present study is reported to promote analgesic effects [19]. The anode electrode was connected to GB 30 (the lateral side of the thigh, proximal to the crushed zone, “proximal” needle) and the cathode electrode was connected to GB 34 (between the crushed zone and distal end of the sciatic nerve, “distal” needle). This disposition was considered the most appropriate for an accelerated regeneration [10]. For the control group, mice were anaesthetized for 15 minutes without acupuncture needles. In a study on peripheral nerve regeneration [10], no differences were reported between controls without needles and those having implanted needles without electricity. We did not stimulate non-acupoints because this generates a transcutaneous electrical stimulation, known to promote analgesia [20] and to interfere with nerve regeneration [9].

Following nerve crush (day post operation, DPO = 0), EA application for group 1 was performed at DPO 5, 7, 9 and 11, i.e. during the period of sensory-motor recovery. For group 2, EA application was performed at DPO 21, 23, 25 and 27, i.e. during the period of pain development.

### Functional tests

Behavioural responsiveness of the mice was tested following one week of habituation to the testing environment and the observer. Two baseline measurements were taken on two separate days preceding the surgery. The mice were then tested every two days after surgery for 6 weeks.

#### Mechanical withdrawal thresholds

The paw withdrawal threshold in response to a mechanical stimulus was measured every two days using a series of graded von Frey filaments. Pressure applied ranged from 0.008 to 4 g beginning with the minimum intensity filament giving a positive response in the scale range for any animal. An ‘up-down’ method was then applied to determine the 50% withdrawal threshold, T50 [21]. According to Dixon [22], optimal threshold calculation by this method requires six responses in the immediate vicinity of the 50% estimated threshold.

##### Thermo-nociceptive testing

Nociceptive threshold to acute thermal stimulation was measured using the paw retrieval test. Focused light from a 12.5 W projection bulb was applied to the middle of the plantar surface of the hind paw (3 mm diameter) [23]. The projection bulb was turned off as soon as the mouse removed its paw, and a digital timer connected in series measured the paw withdrawal latency to an accuracy of 0.1 s. We used a cut-off latency of 15 s to avoid the possibility of tissue damage.

##### Walking track analysis

To assess sensorimotor functional recovery, we used the CatWalk method for both static and dynamic gait analysis [14]. Briefly, animals crossing a walkway with a glass floor are videotaped using a computer-assisted setup and digitized data of paw-floor contact area are used for off-line analysis. One week prior to the left sciatic nerve crush, mice were trained daily to cross the walkway. A normal run was defined as: the mouse crossing the walkway without any interruptions or hitches, the presence of footfall patterns for all four pads, and a running time of around one second to cover 45 cm. Three consecutive runs were recorded. A run in which only three pads were used (i.e. the left hind appears without any recorded trace on the foot floor) was not considered as a “normal” run. In the present study, only mice displaying a normal run were analysed. Gait was monitored every two days. Static parameters (intensity of the paw prints, print width and print length) and dynamic parameters (stance phase, swing phase, swing speed and duty cycle) were measured using CatWalk software 7.1 (Noldus Information Technology, St Louis, France). To eliminate any contribution of weight to the effects observed on the CatWalk, data are expressed as a percentage of respective contralateral values (i.e. compared to the right hind paw).

### Statistics

Results are presented as mean ± SEM. For comparison between the two groups, the data were analysed by two-way ANOVA followed by Tukey’s post-hoc test. The *X*^2^ test was used for run analysis. A Mann–Whitney *U* test was used to compare independent values between groups. A p value < 0.05 was considered statistically significant.

To analyse any possible correlations between the sensory tests and the CatWalk method, a Pearson correlation test was performed.

## Results

### Sensory analysis

**Figure 1:**
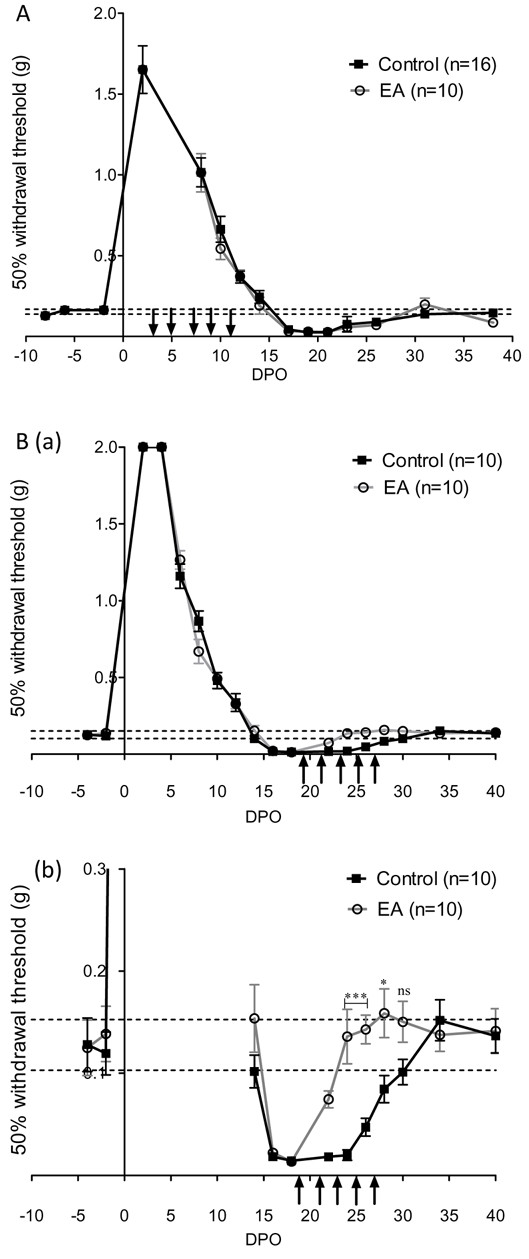
**Analysis of mechanical sensitivity recovery.** (**A**) Time for recovery of sensitivity to a mechanical stimulus using von Frey filaments. The withdrawal threshold (T50) is shown before and after left sciatic nerve crush injury. Vertical arrows indicate electro-acupuncture (EA) application, every two days for 15 min from 3 to 11 DPO (group 1). n is the number of mice in each group; values of T50 for the two groups were pooled before crush injury (n = 26). (DPO: day post operation). (**B**) (a) Effects of electro-acupuncture applied at the moment of pain development from 19 to 27 DPO (group 2). In (b) enlarged view of the period of pain-related symptoms shown in (**a**). * p < 0.05, *** p < 0.001 (ANOVA).

**Figure 2:**
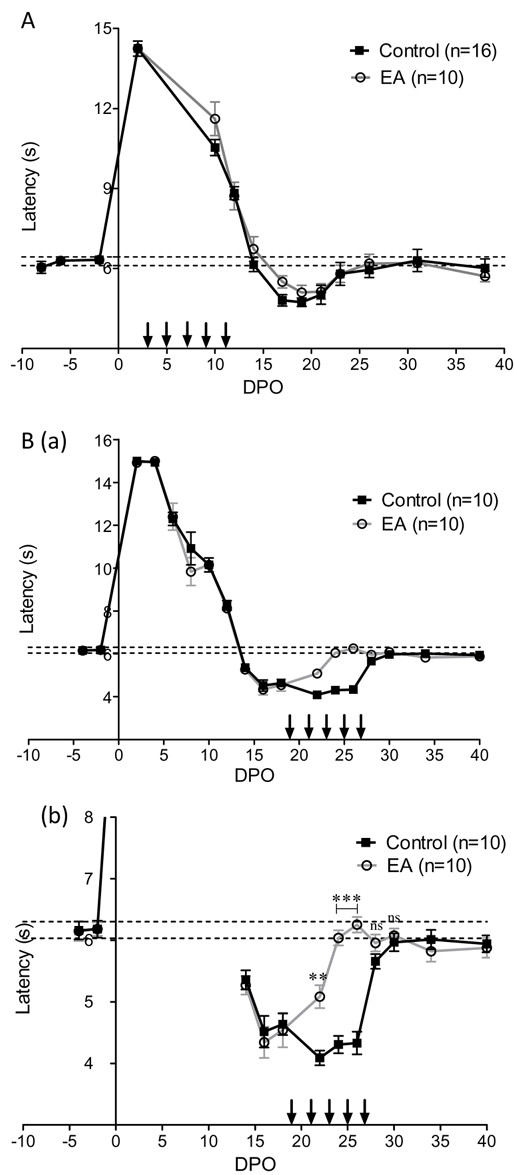
**Analysis of heat sensitivity recovery.** (**A**) Time for recovery of sensitivity to a heat stimulus. The latency of paw withdrawal was measured before and after left sciatic nerve crush injury. Vertical arrows indicate electro-acupuncture (EA) application (group 1). (**B**) (**a**) Effects of electro-acupuncture applied at the moment of pain development (19 to 27 DPO) (group 2). In (**b**) enlarged view of the period of pain-related symptoms. * p < 0.05, ** p < 0.01 (ANOVA).

In a first series of experiments, following crush of the left sciatic nerve, we investigated the recovery of sensory functions in 16 control mice and 10 mice treated with electro-acupuncture (group 1). Recovery of sensory function was assessed by measuring the paw withdrawal threshold in response to mechanical stimulus and the paw withdrawal latency in response to heat stimulus. Before nerve injury, the response to mechanical stimulation with von Frey filaments, T50 was 0.153 ± 0.007 g, n = 26 and the latency for paw retrieval from radiant heat was 6.2 ± 0.1 sec, n = 26. The sciatic crush injury resulted in a transient loss of the withdrawal response to both mechanical and heat stimuli that were progressively recovered over 14–16 days (Figures 1A and 2A). Both mechanical allodynia and heat hypersensitivity become apparent following this period of functional regeneration with a peak around 17 to 22 days post operation (DPO) (Figures 1A and 2A). Recovery of normal sensitivities to mechanical and heat stimuli occurred at 30 DPO following sciatic nerve crush (Figures 1A and 2A). Early treatment with electro-acupuncture, i.e. between 3 to 11 DPO, resulted in no difference in recovery time, nor did it affect the development of mechanical and heat-induced pain.

In a second series of experiments on 10 control mice and 10 electro-acupuncture-treated mice (group 2), we investigated the effects of electro-acupuncture when mechanical and heat thresholds reached values below the control. As observed in group 1, the withdrawal response to both mechanical and heat stimuli progressively recovered over 14–16 days after nerve injury, followed by a period of hypersensitivity. Hypersensitivity was characterised by a lower withdrawal threshold (Figure 1B(a-b)) and a shorter latency to heat response (Figure 2B(a-b) than the initial value recorded between 16 to 28 DPO. These effects demonstrate that motor recovery is accompanied by a transient period of pain-related behaviour. Then, 10 mice were treated with electro-acupuncture every two days throughout the period of pain development (Figures 1Ba, b and 2Ba, b). Under this condition, mice treated with electro-acupuncture showed significantly reduced pain-related behaviour as withdrawal threshold was significantly higher and latency longer than control animals at the time of hypersensitivity development between 20 to 28 DPO. From 30 DPO, no significant differences were observed between control and electro-acupuncture-treated mice.

### Sensorimotor analysis

**Figure 3:**
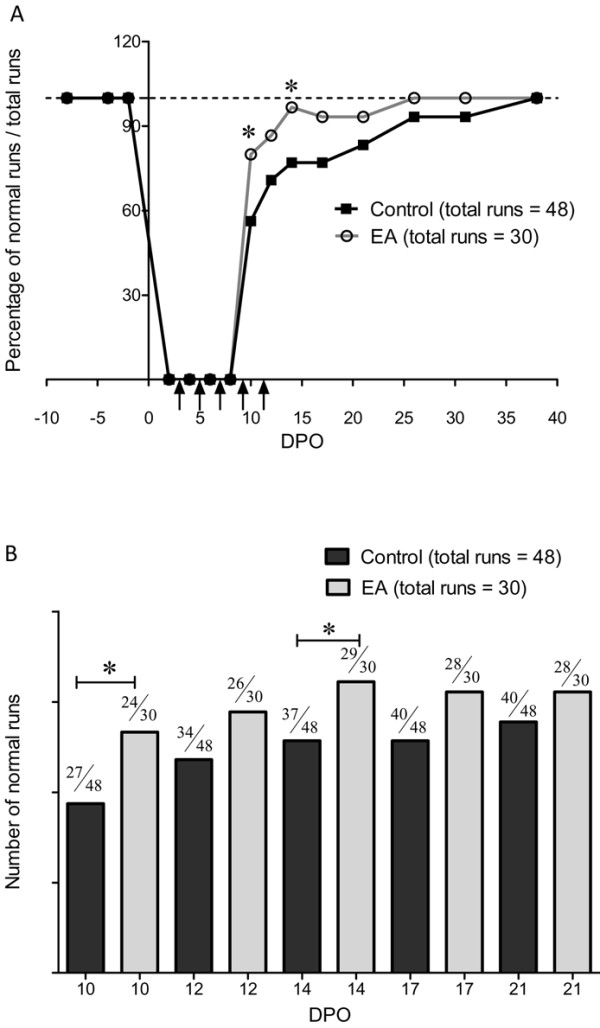
**Analysis of walking runs recovery.** (**A**) Time course of the number of normal runs after left sciatic nerve crush in control and electro-acupuncture (EA) groups. Each mouse was tested for three runs. Graph shows the total percentage of runs tested with 16 control mice and 10 electro-acupuncture-treated mice at 10, 12, 14, 17, 21, 26, 31 and 38 DPO (group 1). Vertical arrows indicate electro-acupuncture application. (**B**) Bar graph representation of the number of normal runs between 10 and 21 DPO. * p < 0.05 (*X*^2^ test).

The effects of early treatment with electro-acupuncture were also tested using the Catwalk test to evaluate both sensory and motor recovery. For comparison, the mice of group 1 used for sensory testing were tested. Firstly, we assessed the global mouse gait by analysing the percentage of normal runs from just after sciatic nerve crush up until full recovery. Figure 3 shows that 1 week after the crush, roughly 60% of control animals displayed normal runs (see methods) and after 5 weeks all mice displayed normal runs. Electro-acupuncture significantly increased the percentage of normal runs one week after the crush to 80%. However, the time to full recovery was not significantly different between the two groups.

**Figure 4:**
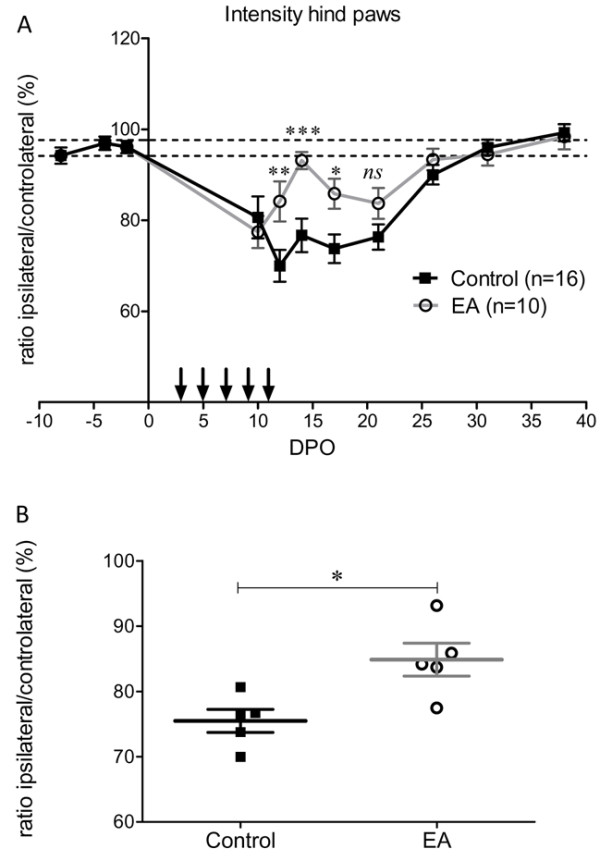
**Analysis of hind paw intensity.** (**A**) Walking-track analysis of the intensity of the injured hind paw print, expressed in relation to that of the controlateral hind paw print before and after left sciatic nerve crush. Vertical arrows indicate electro-acupuncture (EA) application (group 1). * p < 0.05, *** p < 0.001 (ANOVA). (**B**) Global analysis of intensity during the plateau phase (10 to 26 DPO) shows an overall significant increase in paw print intensity following electro-acupuncture. * p < 0.05, *U*-test).

**Figure 5:**
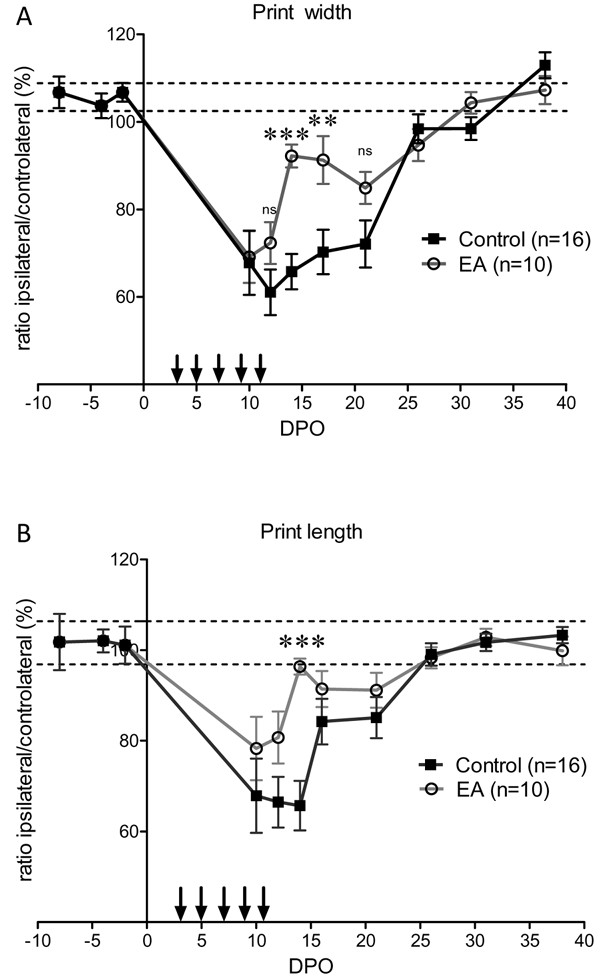
**Analysis of individual parameters of paw print.** (**A**) Print width is the width of the artificial box positioned around the paw print by the CatWalk software. Paw print could be recorded from 10 DPO. The width of the injured paw print was roughly 60–70% of normal value for several days, and rapidly recovered to normal values after 21 DPO. Print width of electro-acupuncture (EA) treated mice was significantly larger at 14 and 17 DPO. Total time for recovery was similar to control mice. (**B**) Print length is the length of the artificial box which the CatWalk software positions around the paw print. The print length of the injured paw was 65% of the normal value from 10–14 DPO and then rapidly recovered to a normal value at 26 DPO. The print length of electro-acupuncture treated mice showed the same length of time for a full recovery as control mice, but displayed a significant apparent and transient recovery at 14 DPO. ** p < 0.01, *** p < 0.001 (ANOVA).

Following sciatic nerve crush, an injured hind paw print of decreased intensity could be assessed from the tenth DPO. Before that, no footprint of the injured hind paw was captured due to mice not using this paw. From 10–26 DPO, there was a plateau phase followed by a complete recovery at 30 DPO (Figure 4A). Electro-acupuncture induced a complex pattern of recovery over time. There was an initial rapid recovery that reached normal values at 15 DPO, followed by a second period of partial loss of paw print intensity between 17–21 DPO. Full recovery was reached at 26 DPO. Analysis of the mean print intensity on 5 days during the plateau phase (10, 12, 14, 17 and 21 DPO) also revealed significant effects of the electro-acupuncture. During this period, the mean paw print intensity of the injured left hind paw relative to the contralateral hind paw was 75.5 ± 1.7% and 84.8 ± 2.5%, n = 5, for control mice and electro-acupuncture-treated mice, respectively, p < 0.05 (Figure 4B). In control mice, the individual paw parameters related to the size of the left hind paw prints were reduced by roughly 40% compared to the contralateral paw print after sciatic nerve injury. Print width and print length remained at this level for roughly 3 weeks before a full recovery at 30 DPO (Figure 5). In electro-acupuncture-treated mice, the print width was initially the same as control mice, i.e. reduced by 40% compared to the contralateral paw at 10 DPO. This was followed by a rapid phase of recovery starting at 12 DPO that reached a level that was just 10% less than the contralateral paw at 14–17 DPO. This represented a significant improvement compared to the 40% reduction still observed in the control mice at the same time point (p < 0.001 at 14 DPO and p < 0.01 at 17 DPO, Figure 5A). The same time course was observed for the print length with a somewhat increased recovery over the same period (p < 0.001 at 17 DPO, Figure 5B). Full recovery was achieved at 30 DPO for both groups.

**Figure 6:**
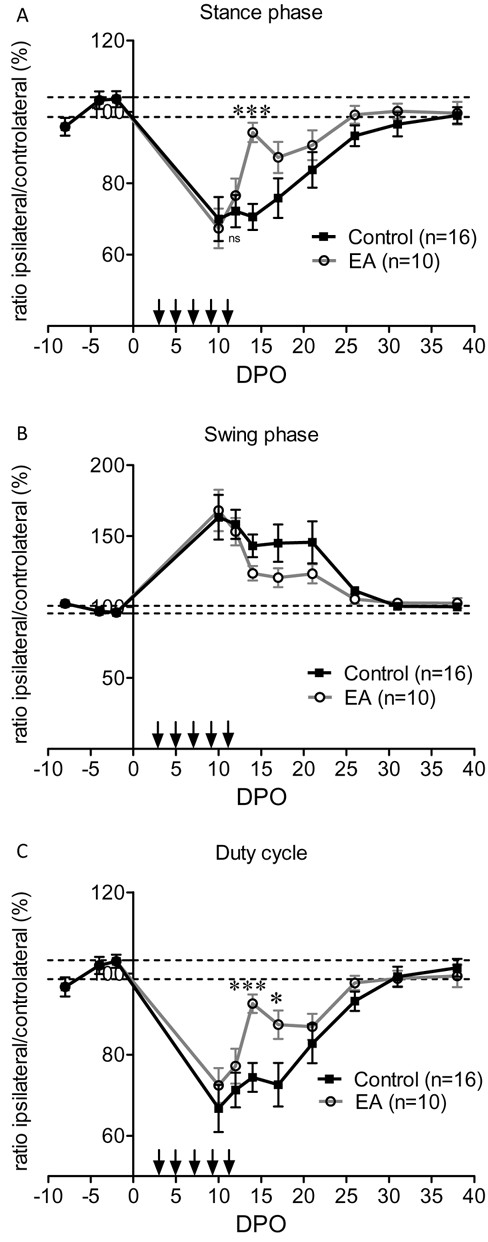
**Analysis of dynamic parameters of gait.** (**A**) Duration of the stance phase (period in which the paw is in contact with the floor during each step cycle). Electro-acupuncture (EA) induced a significant recovery at 14 DPO; time for full recovery was not modified (between 31–38 DPO). (**B**) Duration of the swing phase (period in which the paw is not in contact with the floor during each step cycle). Electro-acupuncture did not significantly affect this parameter at any time. (**C**) Duty cycle (the stance phase/swing phase + stance phase) showed a full recovery between 31–38 DPO in control mice. Electro-acupuncture induced a transient recovery at 14 and 17 DPO. Time for full recovery was not changed. * p < 0.05, *** p < 0.001 (ANOVA).

Analysis of the dynamic parameters showed that in control mice, the stance phase (the duration of contact of the injured paw with the glass plate in a step cycle) was reduced by around 50% of the pre-operative value for 2–3 weeks before a full recovery at 5 weeks (Figure 6A). The swing phase (the duration of no contact of a paw with the glass in a step cycle) mirrored the evolution of the stance phase (Figure 6B). Following electro-acupuncture, the stance phase and the swing phase tended to recover earlier at 2 weeks, with a significant effect at 14 DPO on the stance phase (Figures 6A, B). Analysis of the duty cycle (the percentage of stance phase compared to the total duration of a step cycle) for the left hind paw confirmed the tendency of electro-acupuncture to improve the contact of the paw with the floor, with a significantly reduced recovery time to 14–17 DPO (Figure 6C).

### Correlation between the sensory tests and CatWalk parameters

To analyse the degree of correlation between the sensory tests and the CatWalk method, we determined, in group 1, the Pearson correlation coefficient (p.c.c.) from the period of pain development, 17 DPO, until full recovery of sensorimotor function (38 DPO). Strong correlations (p.c.c. > 0.9) were found in control mice between the two static and one dynamic parameters of the CatWalk method and mechanical sensitivity (Table 1) or heat detection (Table 2). A loss of this correlation was clearly observed in mice treated during the first two weeks with electro-acupuncture (p.c.c. of 0.6-0.7 for mechanical sensitivity (see Table 1) and reduced to 0.5 for heat sensitivity (see Table 2)).

**Table 1:** Pearson coefficient between the von Frey data and the CatWalk parameters

**Parameter of Catwalk vs.**	**n**	**Intensity**	**Print width**	**Duty cycle**
Von Frey in control mice	16	0.979237	0.977687	0.915548763
Von Frey in EA-treated mice	10	0.62321	0.731066	0.718410937

**Table 2:** Pearson coefficient between time latency of the Hargreaves test and the CatWalk parameters

**Parameter of Catwalk**	**n**	**Intensity**	**Print width**	**Duty cycle**
Hargreaves in control mice	16	0.957767	0.905113	0.952181525
Hargreaves in EA-treated mice	10	0.489753	0.105968	0.58584809

## Discussion

To our knowledge, this is the first time that the effects of electro-acupuncture on functional sciatic nerve regeneration have been evaluated in a mouse model. Using sensory tests together with walking-track analysis, we have shown that the sciatic nerve crush in mice induces a loss of sensory and motor functions that recover to normal values between 5–6 weeks. Such rapid recovery has previously been reported in a comparative study between rats and mice [24]. In addition, specific analysis of mechanical and heat sensitivity revealed that recovery of normal sensitivity occurs in 2 weeks, but is then followed by the development of neuropathic pain. Crush-induced neuropathic pain has been observed in the rat and was correlated with the appearance of c-F0s, a known injury marker in the dorsal horn [25]. Similar results have been described following sciatic nerve crush in mice and, as in the present study, showed the appearance of mechanical allodynia two weeks after sciatic nerve crush [24]. In this model of crush injury, it therefore seems that neuropathic pain occurs following the period of regeneration when re-innervation of the paw has been established. Application of electro-acupuncture during the recovery phase of tactile and heat sensitivity (the first 2 weeks following injury), did not modify the time course of recovery or the development of hyperalgesia and allodynia. However, when electro-acupuncture was applied at the moment of neuropathic pain-like symptoms, i.e. from the third to fifth week following injury, we observed a significant recovery to normal levels of sensitivity to mechanical and heat stimuli. These results are in agreement with the known effects of electro-acupuncture in the treatment of pain and demonstrate its potential efficiency on neuropathic pain that develops following a nerve injury.

Numerous studies have aimed to identify the cellular modifications leading to pain relief under acupuncture. Acupuncture induces an increase in the molecules involved in the inhibitory control of pain processing, such as opioid peptides, GABA and somatostatin [15]. While we did not perform an analysis of molecular markers related to pain, the short term effects of electro-acupuncture seems to correlate more with an anaesthetic-like effect rather than to transcriptional remodelling. Consistent with this hypothesis, it has been shown that analgesia induced with a low frequency electro-acupuncture of 2 Hz is associated with the rapid release of β-endorphin and met-enkephalin [19]. Recently, it was demonstrated that adenosine release under acupuncture contributes to local anti-nociceptive effects [26].

Gait analysis using the CatWalk method has recently been validated in rats to evaluate the function of both sensory and motor fibres during a specialized pattern of movement [13,27]. Notably, a good temporal correlation was observed between paw print intensity and the classical static sciatic index (SSI) [14]. In the present study, we have shown with gait analysis, that under our experimental conditions, mice needed 5 weeks to recover normal paw pressure. The same time course was observed for all dynamic parameters tested. Electro-acupuncture significantly increased the percentage of normal runs that could be ascribed to an increased recovery in motor fibres. Both paw print intensity and individual paw and dynamic parameters are in agreement with an increased motor recovery two weeks following nerve crush. Such a preferential effect of electro-acupuncture on motor recovery is reminiscent of data showing that electrical stimulation can promote sensory fibre regeneration but, contrary to motor fibres, only when short duration stimulations are used [8]. Thus, depending on the mode of electrical stimulation, motor and/or sensory fibres are differentially affected.

The molecular mechanisms sustaining motor recovery under electro-acupuncture are poorly identified. However, they could be the same as those involved in the analgesic effects. Indeed, it has been demonstrated that activation of the opioid system enhances nerve regeneration [16]. Cellular changes in chloride homeostasis suggest that activation of GABA-A receptors could also promote nerve regeneration [28] and, within a narrow range of concentration, nitric oxide production under electro-acupuncture could contribute to motor recovery [29,30].

The importance of using specific sensory tests in addition to gait analysis was clearly demonstrated once we applied electro-acupuncture. Indeed, while we showed that electro-acupuncture applied during the first two weeks does not prevent pain development, it did lead to a rather complex pattern of effects on gait. Following the period of accelerated motor recovery induced by electro-acupuncture, our data show that the apparent loss of recovery of static and dynamic gait parameters correlates with the occurrence of pain. This observation is supported by the analysis of the Pearson coefficient correlation between sensory tests and the CatWalk method showing a strong correlation in the control group. However, a clear divergence is observed when electro-acupuncture is applied. This divergence likely reflects the integration of both sensory and motor patterns by the CatWalk method and the fact that electro-acupuncture has no effects on sensory recovery. It should be noted that, depending on the animal model of pain, positive correlations between the von Frey test and CatWalk parameters are either confirmed [13,27], or disputed [31].

In recent years, our knowledge about the cellular and molecular requirements for peripheral nerve regeneration has greatly increased [3]. Nonetheless, obtaining a functional motor recovery remains a challenge. Electrical stimulation of the cut and regenerating nerve fibres promotes motoneuron regeneration but cannot be used in patients [6,7]. Reconstructive strategies following nerve sectioning are commonly used in patients with large nerve injury. These include autologous nerve grafting, silicone guidance tubes [32] and acellular nerve allografts [33]. Cell-based therapy with mesenchymental stem cells appear a promising strategy to create a favourable environment for peripheral nerve regeneration [34]. The electro-acupuncture cannot substitute the above-mentioned strategies but could be used as a complementary approach to stimulate intrinsic motor fibres regrowth properties in patients.

## Conclusions

This work indicates that the simultaneous use of sensory tests and the CatWalk test provides a more detailed picture of functional sciatic nerve recovery, which is particularly important when assessing new therapeutic approaches. In addition, electro-acupuncture appears to be a valuable method to accelerate motor recovery and alleviate neuropathic pain symptoms that occur after nerve crush.

## Abbreviations

DPO: Day post operation; Pcc: Pearson coefficient correlation; T50: 50% paw withdrawal threshold.

## Competing interests

The authors declare that they have no competing interests.

## Authors’ contributions

NSH, VS and FS conceived the study. NSH, CS and VS carried out the experiments. NSH, JV, VS and FS participated in designing the experiments. VS and FS wrote the manuscript. All authors read and approved the final manuscript.

## Pre-publication history

The pre-publication history for this paper can be accessed here:

http://www.biomedcentral.com/1472-6882/12/141/prepub
